# The Impact of Customer Psychological Price on Audit Pricing in the Start-Up Company Market

**DOI:** 10.3389/fpsyg.2020.01562

**Published:** 2020-07-02

**Authors:** Rui Ding

**Affiliations:** School of Economics and Management, Nanjing University of Science and Technology, Nanjing, China

**Keywords:** product market, customer psychological price, audit pricing, startup company, psychological expectations

## Abstract

Start-up companies are confronted with various risks and many uncertainties, and professional auditing can fully analyze start-up companies. In this way, both parties may maximize their interests through auditing the market activities. Based on the characteristics of start-up companies, this study explores the impact of customer psychological prices on audit pricing. The impact of customer psychological prices on audit pricing decisions was systematically analyzed from various angles, thereby determining whether it will affect the development of the product market. The results show that product market dominance reduces the agency costs between the owner and manager of the customer company. In other words, if the customers have greater control over the product market, they will have a lower business risk, and the auditor risk will be reduced accordingly, hence a lower audit fee. In the start-up company market, even if the financing dilemma restricts the survival and development of the company, customers still have psychological expectations for audit pricing. When their psychological price of products is different from the market price of products, it may affect the market advantage of products with lower audit fees, and further expands the previous research. In the market, customers also have certain psychological expectations for “auditing” products. Therefore, strengthening the relationship between auditors’ pricing and customers’ psychological prices has a positive effect on enhancing the competitiveness of product markets, which also increases the operating efficiency of start-up companies.

## Introduction

Since China joined the World Trade Organization (WTO) in 2001, Chinese companies have not only faced competition from the domestic market but also participated in the fierce competition in the international market (Tsao et al., 2017). Increased globalization and reduced trade barriers have intensified the competition in product markets. At the same time, the world financial crisis in 2008 caused huge changes in the market structure of various industries around the world. Some scholars have studied whether the market competitiveness of products is related to audit pricing (Chang and Wong, 2018). Scholars believe that companies with competitive advantages in product market power can continue operations and set up barriers for potential new entrants, thereby reducing agency costs between managers and shareholders. At the initial stage of their life cycle, start-up companies do not have adequate funds and resources, leading to a low market share, small production scale, insufficient profitability, and certain financial difficulties. In this case, professional audit services can issue reasonable audit report opinions on the financial statements of the companies, avoiding the risk of lawsuits of start-up companies (Su et al., 2019).

Chhaochharia et al. (2009) explored the role of market competitiveness of products in the management of listed companies in the United States and the differences in shareholders’ interests; the results showed that the market competitive advantage of products significantly reduced agency conflicts. According to agency cost theory, product market competition, as a supervision mechanism, makes auditors face the pressure to reduce audit costs by reducing audit time. At the same time, some studies have shown that fierce product market competition not only increases the business risk of auditors but also increases the business risk of customers. Auditors are responsible for discovering material misstatements and confidently issuing audited financial statements (Yu, 2020). If auditors cannot use standard audit procedures to reduce the risk of customer breaches or errors to acceptable levels, they will require payment of insurance premiums to compensate. The auditors judge the audit risks by the operating financial statuses of clients. Poor financial status indicates a high probability of audit risk. The auditors need to obtain more sufficient and higher-quality evidence to control the inspection risk. Therefore, the audit cost is higher. Studies have also confirmed that effective governance mechanisms of enterprises will reduce the audit risks of listed enterprises and companies assessed by auditors, thereby charging lower audit fees.

The audit pricing strategy of a company is a key issue often considered in the marketing field. When a company highlights a new product, the company will price it and may need to adjust the product price as the market changes. These are the basic issues to be considered in the marketing process (Choi et al., 2019). However, many companies may ignore the impact of customer behavior and customer psychological factors during auditing pricing and marketing planning, which may indirectly affect the sales and profits of the company. Therefore, the auditor will predict the profitability by assessing the customer’s risk. To reduce potential losses, the audit company will increase the audit time and require the risk premium to compensate by increasing the audit cost (Lemmerer and Menrad, 2019).

Although the competitiveness of the industry is the same as all companies in the same industry, companies with stronger product market strength may protect themselves from the effects of fierce competition. By using the estimated profit-cost-margin (EPCM) values, this study follows previous research to measure the specific product market strength of a company in the industry. In this study, the existing research is extended to a developing country market such as China. From the perspective of customer psychology, the relationship between product market competitiveness and audit pricing under different institutional backgrounds is explored. This study aims to expand the information of customers on the establishment of audit prices, systematically analyze the impact of customer psychological prices on company pricing decisions from various angles, and enrich the role of customer bargaining power in the relationship between product market power and audit pricing.

## Literature Review

Traditional audit pricing theory believes that in the competitive audit market, both auditors and clients should consider audit costs and audit risks during the negotiation of audit pricing. Most of the research on the influencing factors of audit pricing have selected the company size as the explanatory variable of audit pricing, and all have confirmed that the company size has the strongest explanatory power to audit pricing (Walsh et al., 2016). The influencing factors of audit pricing have always been an important topic in the field of company finance and audit. Mou et al. (2014) analyzed the ways that executive rights and internal control systems influence audit fees. The results suggested that they mainly affect the audit workload and audit risk, and then, audit costs (Mou et al., 2014). Shi et al. (2015) explored how analysts, who are also external corporate governance mechanisms, influence the decision-making of auditors. The results have confirmed that the firm’s audit pricing will refer to the analyst’s forecast. The more accurate the analyst’s forecast is, the lower the audit fees auditors charge; on the contrary, the more discrete the analysts predict, the higher the audit fees are.

In addition to the company size and governance, audit pricing is also affected by customers’ psychological expectations of audit quality. Some scholars pointed out that the customer’s demand for audit quality depends on their motivation to choose audit and the firm’s industry expertise; at the same time, they also discussed the impact on audit quality from the legal system environment faced by auditors (Defond and Zhang, 2014). The entry or exit of an accounting firm in the market, or the changes in clients’ demand for audit quality, will bring about changes in the supply and demand relationship of the audit market, while this change will also affect the audit pricing and audit quality of the firm (Rand et al., 2017). Regardless of whether the auditing fee charged by the firm for auditing services is reduced, the accounting firm will reduce transaction costs with listed companies as much as possible. The reason is that on the one hand, the reduction in transaction costs may make the firm issue lower audit pricing to attract customers; on the other hand, with the same audit fees, the firm will obtain more service premium and profit space.

In summary, there are many studies on the factors affecting audit pricing in this field, and their respective focuses are also different, including company size, audit quality, and market competition. However, as an important party of auditing process, customers also have a very important influence on audit pricing. Therefore, from the perspective of customers’ psychological prices, the correlation between product market competitiveness and audit pricing under different institutional backgrounds is explored.

## Materials and Methods

### Audit Pricing and Market Competition

Auditing is a commodity and the result of auditing accounting statements. Currently, for the relationship between audit report products and non-audit report products, there are two main views theoretically. One is that the relationship between the two types of audit products is irreconcilable; the audit report product reflects the essential content of the audit. If the auditor provides non-audit report products at the same time, it will lead to loss of independence and reduce the quality of audit products. Another view holds that the relationship between the two types of audit products can be reconciled; the auditor can provide non-audit report products at the same time. If the auditor is professional enough, the audit quality will not be affected. In a market environment, an accounting firm can be regarded as a party providing audit services, and a listed company can be regarded as a party receiving audit services. Both parties have their goals in market activities and maximize their interests through audits (Pleman et al., 2019). The audit pricing formulated by external audit institutions not only includes audit costs but also is affected by the macro environment, audit risks, and market structure of the company. The specific audit account can reflect the risks and benefits in the audit process. The accounting firm audits the financial statements, operating risks, and internal control of listed companies, and issues audit reports with legal benefits. This credible audit report can effectively balance the information asymmetry between companies and investors, thereby improving the investment efficiency of investors. Regional audit pricing based on audit risk requires the consideration of the impact of regional differences on audit risk and the risk compensation in audit pricing. Research shows that audit pricing reflects significant misstatement risks (Price et al., 2019).

The audit market refers to a special transaction environment formed by the “audit” business. The main bodies of the two parties to the transaction are listed companies and accounting firms. In any market, the price system is an important factor in maintaining the balance of interests of all parties to the transaction. Price is also one of the key factors that determine the characteristics of the market structure. The premise of the orderly operation of the audit market is to have a complete audit system to provide protection, thereby regulating the behavior of the accounting firm and the individual auditor. Product market power symbolizes the comparative ability of a company to determine prices in the market by manipulating the levels of supply, demand, or both (Kushlev et al., 2019). Therefore, companies with stronger product market power may fully utilize their advantages by manipulating market prices and increase profit cost ratios while setting barriers for potential new entrants. In the field of microeconomics, some scholars have proposed a product market power measurement method. The excess price cost margin is used to describe the monopoly capacity of a product and then measure the product market strength of a company compared to its peers.

In recent years, with the continuous development of the accounting firms in China, the concentration of the audit market has increased, and studies have confirmed that the concentration of the audit market will increase due to the intensification of market competition (Kudryavtsev, 2019). On the one hand, there is fierce competition in the audit market; on the other hand, there is local monopoly in large accounting firms. The fierce competition in the audit market will not affect the independence of audit work, which means that it will not cause a decline in audit quality. In addition, as China’s government and society continue to increase audit supervision, the concentration of the audit market has declined. At this time, the audit quality has improved significantly, demonstrating a negative correlation between the audit market concentration and audit quality.

### Impact of Customer Psychological Price on Audit Pricing in Start-up Companies

**FIGURE 1:**
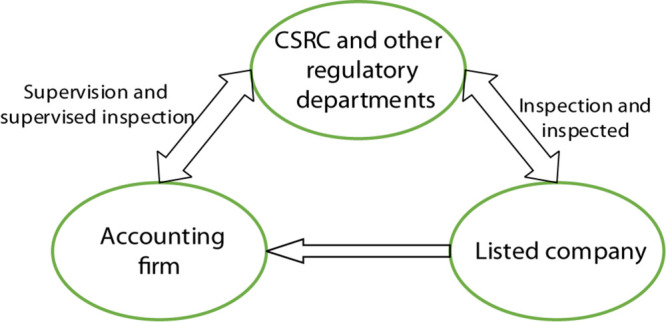
Tripartite game approach to audit transactions.

Due to the limited funds and few financing channels, the size of start-up companies is limited inevitably, which will lead to slower business development and lower product market share. Audit services can maintain the financial laws and regulations of the companies, improve business management, and increase economic efficiency. Generally, once problems are found during the auditing process, improvement opinions will be received in time to help the company make financial statements, which is of vital importance to the legal and positive development of the company. In the process of audit pricing, listed companies as customers who receive audit services, their psychological prices will affect the final audit pricing. Audit pricing is a common concern of the company’s internal financial and auditing industries, which is affected by various company characteristics such as company size and property rights. Customers will also set their own audit pricing according to their business development and operation, so there is a pricing game between the psychological price of customers and the audit pricing of accounting firms (Bills et al., 2018). The tripartite game participation methods, including the regulators such as the China Securities Regulatory Commission and the China Investment Association, are shown in Figure 1.

The joining of regulatory agencies such as the Securities Regulatory Commission will further review listed companies based on audit reports provided by accounting firms. In the tripartite game model, a listed company and accounting firm are two elements that seek to maximize benefits in the cooperation process (Huang et al., 2019). Therefore, a reasonable audit price is the basic guarantee for ongoing transactions between the two parties. From the perspective of the company, if internal governance is unreasonable, it will lead to higher agency costs within the company and require auditors to assess the risk of major misstatement of the company. When assessing a company with a large audit risk, the auditor needs to pay more for the audit. It needs to increase audit efforts, expand the scope of the audit, and increase the audit processes. Therefore, the audit resources invested by the accounting firm will increase significantly. Correspondingly, as audit costs increase, audit pricing will increase (Sulistiyo and Ghozali, 2017). In terms of auditing this market service, the accounting firm is also an independent business organization. When receiving each audit business, the accounting firm will negotiate with the company for the specific audit service and audit pricing. In addition, the final audit pricing of the accounting firm needs to include the cost of company information collection, which is also a factor in the pricing differences between the two parties.

Auditing services can be seen as a special product on the market. When a company pays for the audit service as a customer, it will usually make a value prediction of the audit service as a commodity based on the service prices of other items that the customer refers to. Moreover, after the real service price is made clear, the service selection is determined and the customer psychological price is formed (Bills et al., 2016; Sari et al., 2019). If the pricing of the audit service exceeds the customer budget price, it is easy to reduce the customer’s desire to choose the service. Therefore, the relevant pricing personnel in the audit market need to price the service after understanding the basic psychological price of the customer, which is more conducive to the economic benefits of the accounting firm.

### Research Hypotheses

Existing research has tested whether the commercial risks of customers have an impact on audit costs. Johnstone has built a model to describe customer acceptance decisions for risk assessment and risk adaptation. The auditor evaluates the customer’s risk and uses their evaluation results to decide on the audit plan to avoid or adapt to the risk (Asmara, 2016). Companies with stronger market competitiveness may set market prices to control their cost-margin and have advantages in setting barriers for potential new market entrants. Therefore, when the product market competition is incorporated into the correlation between customer operating risk and audit costs, it is assumed that customers with greater product market power face lower operating risks, which in turn reduces audit risks and audit costs (Finley et al., 2018).

Studies have confirmed that customers have an impact on service providers’ revenue and revenue realization. In turn, service providers may influence the psychological pricing of customers. For both parties of the audit supply chain, there is a contractual relationship between the auditor and the customer on whether to disclose audit fees. As a result, the third-party supervision of audit fees may exacerbate the game between the two (Kuntari et al., 2017; Krishnan et al., 2019). Since the end of 2001, listed companies in China have been required to disclose audit fees. This move reflects competition between the accounting firm and auditors, and thus expands the number of information that customers have on audit pricing. Therefore, it is necessary to pay attention to the role of audit customer bargaining power in affecting the relationship between product market power and audit costs (Sofia and Avianti, 2019).

Industrial competitiveness is the same for all companies in the same industry, while the market competitiveness of a product represents the market position of a company itself. On the one hand, audit companies may undermine their audit independence and use a “low-profile” strategy in exchange for retaining important customers. On the other hand, due to the large reputational risk capital of large customers, auditing companies tend to provide higher levels of assurance for important customers. Therefore, auditors will increase their auditing efforts and provide differentiated services. At this time, the customer psychological pricing of audit services will have a greater impact on audit pricing (Bik and Hooghiemstra, 2018).

Hypothesis 1: For customers with greater product market influence, the psychological price of the customer will have a greater impact on audit pricing. Audit service providers often charge fewer audit fees to retain important customers and enhance their influence.
Hypothesis 2: When the internal competition in the auditor industry is more intense, the psychological price of customers for audit fees will tend to decrease. At this time, customers with strong competitiveness in the product market will often be charged fewer audit fees.

### Data Source and Model Design

To reduce the huge changes in sample selection after China’s accession to the WTO in 2001, this study selects samples since 2005. The samples include 21,154 company annual observations from 2005 to 2017. First, the listed companies of Growth Enterprise Board are excluded. Because the market competition of listed companies of Growth Enterprise Board is distinct from the market competition of listed companies of the Main Board, the included samples exclude financial companies (using the industry code of the 2012 listed company classification guide issued by the United States Securities and Exchange Commission). Besides, the company’s annual data with missing dependent, independent, and control variable data are excluded. To mitigate the effects of extreme values on the results of this study, all continuous variables are ranked at the head and tail of the 1% level each year.

Based on the existing research, this study selects some control variables that affect audit costs to control the fixed effects: customer company size (SIZE), customer leverage (LEV), financial distress (LOSS), inventory asset ratio (INVREC), return on assets (ROA), property, plant, and equipment asset ratio (PPE), operating cash flow (CASH_FLOW), market to book ratio (MB), and annual dummy variables and industry dummy variables (Shen et al., 2019). The following models are built to test Hypothesis 1:

$$L\,A\,F=\alpha_{0}+\alpha_{1}\,E\,P\,C\,M+\alpha_{2}\,S\,I\,Z\,E+\alpha_{3}\,L\,E\,V+\alpha_{4}\,L\,O\,S\,S$$

(1) $$\begin{array}{l} +{\text{α}}_{\text{5}}INVREC+{\text{α}}_{\text{6}}ROA+{\text{α}}_{7}PPE+{\text{α}}_{8}CASH-FLOW\\ +{\text{α}}_{\text{9}}MB+yeardummies+industrydummies+ \end{array}$$

For the ability of auditors and customers to compete with each other’s psychological prices, the auditor industry competition (HERF_AUDITOR), experts (SPECIALIST), and customer importance (OFFICEIMPORT) are used as moderating variables. The industry competition of auditor (HERF_AUDITOR) is calculated by the relative value of the sum of the squared sums of all customers and audit fees for the same industry in the same year. An expert is an indicator. If the auditor has at least 10% of the total market share of an industry, SPECIALIST is set to 1; otherwise, it is set to 0. Customer importance (OFFICEIMPORT) is a measure of the ratio of customer fees to total fees paid by all public customers to the auditing firm. For customer bargaining power, the Pearson correlation coefficient of the percentage change of industry members is used to test the customer industry homogeneity (HOM). The customer industry competition (LNN) is defined as the number of companies in the same year and industry, and customer business complexity (COMPLEXITY) is also being considered. In the research on customer bargaining power based on their psychological price, the size of the company, company leverage, financial distress, inventory asset ratio, return on assets, property, plant, and equipment asset ratio, operating cash flow, market to book ratio, and annual and industry fixed effects are also controlled (Wu and Wu, 2017; Chen, 2019).

$$L\,A\,F=\beta_{0}+\beta_{1}\,E\,P\,C\,M+\beta_{2}\,H\,E\,R\,F\,\_\,A\,U\,D\,I\,T\,O\,R+\beta_{3}\,S\,P\,E\,C\,I\,A\,L\,I\,S\,T$$

$$+\beta_{4}\,O\,F\,F\,I\,C\,E\,I\,M\,P\,O\,R\,T+\beta_{5}\,H\,O\,M+\beta_{6}\,L\,N\,N$$

$$+\beta_{7}\,C\,O\,M\,P\,L\,E\,X\,I\,T\,Y+\beta_{8}\,S\,I\,Z\,E+\beta_{9}\,L\,E\,V+\beta_{10}\,L\,O\,S\,S$$

$$+\beta_{11}\,I\,N\,V\,R\,E\,C+\beta_{12}\,R\,O\,A+\beta_{13}\,P\,P\,E+\beta_{14}\,C\,A\,S\,H\,\_\,F\,L\,O\,W$$

(2) $$+\beta_{15}\,M\,B+y\,e\,a\,r\,d\,u\,m\,m\,i\,e\,s+i\,n\,d\,u\,s\,t\,r\,y\,d\,u\,m\,m\,i\,e\,s+\varepsilon$$

## Results

### Descriptive Analysis

**FIGURE 2:**
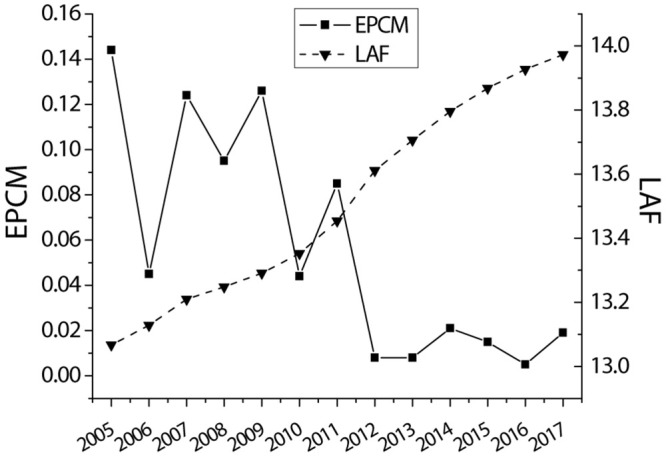
Descriptive statistics of the sample distribution of panel A by year.

**FIGURE 3:**
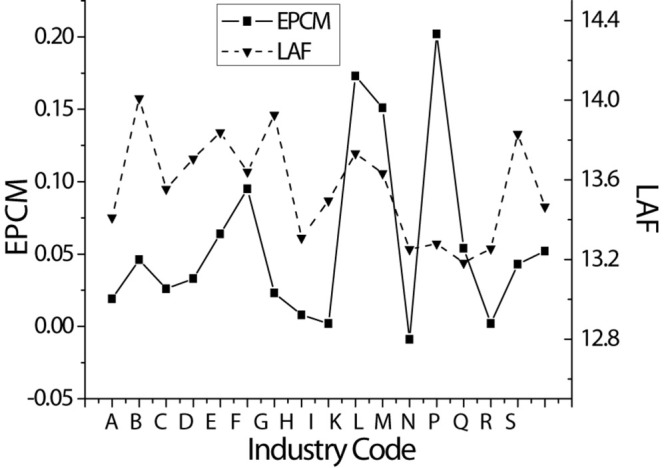
Descriptive statistics of the sample distribution of panel B by industry.

Table 1, Figures 2, 3 provide detailed company sample information by year and industry. A total of 21,154 company annual observation data are obtained from the CSMAR database from 2005 to 2017. Panels A and B list descriptive statistics by year and industry. It is observed that the average audit cost has steadily increased during the sample year; the product market power of the companies in the scientific research and technical service industry is the weakest, and the product market power of the water conservancy, environmental and public facilities management industry companies is the strongest.

**TABLE 1:** Descriptive and correlation statistics.

***Year***	***Frequency***	***EPCM***		***LAF***
2005	845	0.144		13.067
2006	815	0.045		13.128
2007	981	0.124		13.210
2008	1274	0.095		13.248
2009	1357	0.126		13.291
2010	1513	0.044		13.352
2011	1675	0.085		13.454
2012	1983	0.008		13.611
2013	1969	0.008		13.706
2014	1990	0.021		13.796
2015	2087	0.015		13.868
2016	2196	0.005		13.927
2017	2469	0.019		13.973

**Panel B: Statistics by industry**		

**Industry Code**	**Industry**	**Frequency**	***EPCM***	***LAF***

A	Agriculture, forestry, animal husbandry and fishery	306	0.019	13.411
B	Mining	669	0.046	14.010
C	Manufacturing	12763	0.026	13.555
D	Utilities	866	0.033	13.707
E	Construction	622	0.064	13.839
F	Wholesale and retail	1533	0.095	13.641
G	Transportation	829	0.023	13.926
H	Accommodation and catering	110	0.008	13.309
I	Information tech.	959	0.002	13.496
K	Real estate	1265	0.173	13.733
L	Leasing and business services	304	0.151	13.633
M	Scientific research and polytechnic services	135	−0.009	13.251
N	Water conservancy, environment, and public facilities management	241	0.202	13.279
P	Education	47	0.054	13.183
Q	Health and social work	73	0.002	13.255
R	Culture, physical education, and recreation	212	0.043	13.831
S	Others	220	0.052	13.465

**FIGURE 4:**
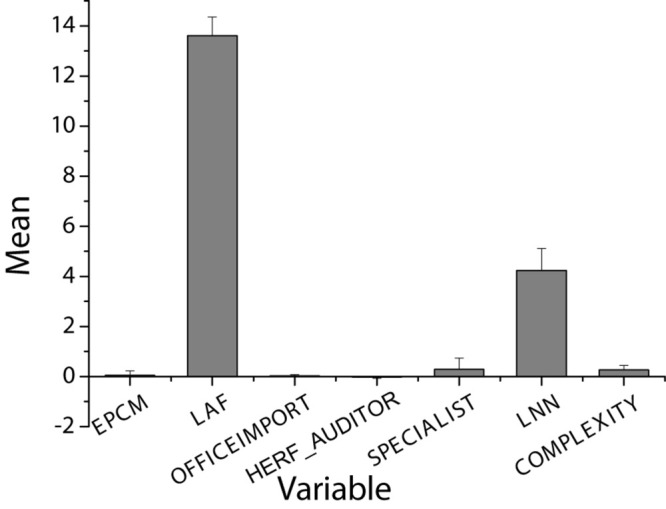
Descriptive statistics of key variables in panel A.

Table 2 and Figure 4 show descriptive statistics of the variables and their correlations. It is observed from panel A that the natural logarithm of audit fees is between 11.918 and 16.524, with an average of 13.605 and a median of 13.487. The fees collected from a single customer account for 2.6% of all customer fees. The relative average of HERF_AUDITOR is 0.024, which is much less than 1, indicating that competition among auditors is very fierce. At the same time, Table 2A also shows that 28.5% of the sample companies hired industry experts.

**TABLE 2A:** Descriptive and correlation statistics of panel A.

**Panel A: Descriptive statistics**							

**Variables**	**Mean**	**sd**	**Min**	**p25**	**Median**	**p75**	**Max**
*EPCM*	0.045	0.182	−0.828	−0.041	0.020	0.108	0.879
*LAF*	13.605	0.751	11.918	13.122	13.487	13.998	16.524
*OFFICEIMPORT*	0.026	0.054	0.002	0.005	0.014	0.032	1.000
*HERF_AUDITOR*	−0.024	0.043	−1.000	−0.026	−0.012	−0.007	−0.003
*SPECIALIST*	0.285	0.451	0	0	0	1.000	1.000
*LNN*	4.238	0.877	0	3.638	4.407	4.942	5.778
*COMPLEXITY*	0.270	0.177	0	0.136	0.244	0.370	0.976
*SIZE*	22.026	1.299	18.865	21.110	21.874	22.774	26.240
*LEV*	0.474	0.215	0.044	0.311	0.475	0.630	1.467
*LOSS*	0.100	0.301	0	0	0	0	1.000
*INVREC*	0.165	0.152	0	0.066	0.127	0.211	0.784
*ROA*	0.036	0.059	−0.436	0.012	0.034	0.063	0.283
*PPE*	0.249	0.176	0.001	0.110	0.215	0.357	0.801
*CASH_FLOW*	0.045	0.076	−0.226	0.004	0.045	0.090	0.291
*MB*	3.938	4.425	−15.936	1.813	2.831	4.620	61.004

**TABLE 2B:** Descriptive and correlation statistics of panel B.

**Panel B: Correlation analysis**										
	**(1)**	**(2)**	**(3)**	**(4)**	**(5)**	**(6)**	**(7)**	**(8)**	**(9)**	**(10)**
**1**-*EPCM*	–	−0.068***	0.054***	−0.184***	−0.344***	0.031***	0.494***	−0.120***	0.240***	0.015**
**2**-*LAF*	−0.063***	–	0.714***	0.210***	−0.044***	−0.021***	0.002	−0.070***	0.020***	−0.182***
**3**-*SIZE*	0.056***	0.763***	–	0.368***	−0.111***	0.019***	–0.001	−0.023***	0.028***	−0.389***
**4**-*LEV*	−0.160***	0.203***	0.329***	–	0.200***	0.226***	−0.435***	0.029***	−0.170***	−0.146***
**5**-*LOSS*	−0.324***	−0.046***	−0.111***	0.224***	−	−0.026***	−0.520***	0.121***	−0.169***	0.019**
**6**-*INVREC*	0.108***	0.003	0.098***	0.273***	−0.033***	–	−0.094***	−0.322***	−0.222***	–0.011
**7**-*ROA*	0.416***	0.030***	0.060***	−0.410***	−0.647***	−0.062***	–	−0.128***	0.374***	0.214***
**8**-*PPE*	−0.096***	−0.027***	0.029***	0.073***	0.125***	−0.396***	−0.141***	–	0.279***	−0.149***
**9**-*CASH_FLOW*	0.177***	0.033***	0.029***	−0.171***	−0.157***	−0.248***	0.338***	0.254***	–	0.049***
**10**-*MB*	−0.079***	−0.130***	−0.302***	–0.007	0.091***	−0.040***	0.051***	−0.096***	–0.004	–

***, **, and * represent significance at the 1, 5, and 10% levels, respectively.

Panel B contains univariate Pearson and Spearman correlations between audit fees, interest variables (EPCM), and other control variables. It is found that companies with stronger product market power often charge lower audit fees. There is a significant correlation between LAF and most control variables. EPCM and the natural logarithm of audit fees are negatively correlated. Therefore, Hypothesis 1 is verified.

### Subsample Regression

Model (2) is used to test Hypothesis 2 from the bargaining power between the auditor and the customer. The subsample regression results are shown in Table 3. When the moderating variables are continuous, the lowest and highest quartiles of these variables are used to compare the differences between the two subgroups. If the moderating variable is a dummy variable, the difference between the two subsamples is tested based on the two values of the dummy variable. Based on the *t*-test and significance level of the two subsamples of the mean, the standard errors of heteroscedasticity are clustered at the company level.

**TABLE 3:** Subsample regression results of each panel.

**Panel A**		

**Dependent variable: *LAF***		

	***LOW_HERF_AUDITOR***	***HIGH_ HERF _AUDITOR***
*EPCM*	−0.235***	−0.539***
	(−3.92)	(−5.41)
Industry Fixed Effects	YES	YES
Year Fixed Effects	YES	YES
SE clustered by firm	YES	YES
N (firm-years)	5289	5289
Adjusted *R*^2^	0.601	0.587
Diff. in coefficients	0.304***	
χ2	7.11	

**Panel B**		

	***NON-SPECIALIST***	***SPECIALIST***

*EPCM*	−0.222***	−0.378***
	(−7.28)	(−5.19)
Industry Fixed Effects	YES	YES
Year Fixed Effects	YES	YES
SE clustered by firm	YES	YES
N (firm-years)	15122	6032
Adjusted *R*^2^	0.636	0.693
Diff. in coefficients	0.156**	
χ2	4.26	

**Panel C**		

	***LOW_LNN***	***HIGH_ LNN***

*EPCM*	−0.235***	−0.539***
	(−4.02)	(−5.35)
Industry Fixed Effects	YES	YES
Year Fixed Effects	YES	YES
SE clustered by firm	YES	YES
N (firm-years)	5289	5289
Adjusted *R*^2^	0.598	0.587
Diff. in coefficients	0.304***	
χ2	6.78	

*** and ** indicates the significance at the 1 and 5% levels, respectively.

Panel A compares the differences in the coefficients of the two subsamples. The EPCM difference coefficients of the two subsamples are 0.304 (*P* < 0.01), which supports that the fiercer the audit competition is, the weaker the auditor’s bargaining power over audit fees is. The moderating effect of different expert values of panel B is compared. The EPCM difference coefficient of the two subsamples is 0.156 (*P* < 0.05), which indicates that customers with stronger market strength are often be charged them fewer audit fees. Panel C compares the different levels of customer competition for the company’s product market power, i.e., the advantage of being charged fewer audit fees. The coefficient of difference between the two subsamples of EPCM is 0.304 (*P* < 0.01), indicating that increased competition in the customer industry will reduce the disciplinary mechanism of company potential risk and further decrease the audit costs.

## Discussion

The product market power symbolizes the comparative ability of a company to determine prices in the market by manipulating the levels of supply, demand, or both. Therefore, a company with a stronger market strength will have a competitive advantage in the product market, and thus bring stable profits to the company (Obschonka et al., 2019). This study found that the competitiveness of the company’s product market is negatively correlated with audit costs, showing that customers with higher product market power have lower operating risks, and the audit risks and audit costs can be reduced. In addition, this study also expands previous research by providing evidence to prove whether the customer’s bargaining power will reduce or strengthen the company’s product market power advantage by being charged fewer audit fees (Wu and Song, 2019). The research results show that the intensification of competition in the audit industry, the auditor industry specification, and the intensification of competition in the customer industry have strengthened the market power advantage of company products, i.e., audit fees will be reduced. The importance of customers to auditors, the homogeneity of customers’ industries, and the complexity of customers’ businesses have weakened the market power advantage of companies that charge fewer audit fees. To ensure that the basic empirical results of this study are robust, first, the alternative measures of product market competitiveness are used to prove that the main results of this study are not limited by specific measures. In an environment where the audit market is oversupplied and there are a large number of accounting firms, most audit service products are considered to be homogeneous. Therefore, accounting firms need to standardize the auditing process and improve their audit quality to gain more competitive advantages. In perfect market competition, the price between accounting firms is equal to the marginal cost, and the profit of the accounting firms is zero. However, considering the differences in products, the quality of audit reports issued by different accounting firms is different. Listed companies have their preferences for the selection of accounting firms. The equilibrium price and marginal cost are different at this time.

This study selects the capital market of China, a developing country, as the research object, and examines the relationship between product market power and audit costs in various industries. In the market, customers also have certain psychological expectations for products. When their psychological price of products is different from the market price of products, it may affect the market advantage of products with lower audit fees, which further expands the previous research (Wu et al., 2020). Strengthening the relationship between auditors’ pricing and customers’ psychological prices has a positive effect on enhancing the competitiveness of product markets. Therefore, it is of positive significance to use the situation of Chinese capital market to verify the relationship between the psychological price of customers and audit pricing in the product market, thereby understanding the customer psychology and give full play to the market advantage of products to the greatest extent (Li et al., 2019).

## Conclusion

Faced with a highly uncertain competitive environment, startup founders will make organizational changes. The founders influence the commitments of their subordinates and strengthen the behaviors of opportunity identification and advantage search, in order to realize the entrepreneurial vision. Under such circumstances, professional auditors will have a preliminary understanding of the company, review whether there are loopholes in internal control, and identify abnormal problems timely after reviewing the financial statements, thereby laying a solid foundation for the stable development of start-up companies. For customers with greater product market influence, the psychological price of the customer will have a greater impact on audit pricing. Audit service providers often charge fewer audit fees to retain important customers and enhance their influence. When the internal competition in the auditor industry is more intense, the psychological price of customers for audit fees will tend to decrease. At this time, customers with strong competitiveness in the product market will often be charged fewer audit fees. Due to the limitation of the data, the author may only choose the listed companies in China as the research object. Because there is no market competitiveness of private companies from the same industry, the measurement of China’s overall product market competitiveness is not comprehensive. In addition, the specific impact of audit pricing and customer psychological prices is not clarified in this study. Therefore, a more in-depth analysis is needed in the subsequent study.

## Data Availability Statement

The raw data supporting the conclusions of this article will be made available by the authors, without undue reservation.

## Ethics Statement

The studies involving human participants were reviewed and approved by the Nanjing University of Science and Technology Ethics Committee. The patients/participants provided their written informed consent to participate in this study.

## Author Contributions

The author confirms being the sole contributor of this work and has approved it for publication.

## Conflict of Interest

The author declares that the research was conducted in the absence of any commercial or financial relationships that could be construed as a potential conflict of interest.

## References

[B1] Asmara R. Y. (2016). Effect of competence and motivation of auditors of the quality of audit: survey on the external auditor registered public accounting firm in Jakarta in Indonesia. *Eur. J. Account. Audit. Finance Res.* 4 43–76.

[B2] Bik O. Hooghiemstra R. (2018). Cultural differences in Auditors’ compliance with audit firm policy on fraud risk assessment procedures. *Auditing J. Pract. Ther.* 37 25–48. 10.2308/ajpt-51998

[B3] Bills K. L. Cunningham L. M. Myers L. A. (2016). Small audit firm membership in associations, networks, and alliances: implications for audit quality and audit fees. *Account. Rev.* 91 767–792. 10.2308/accr-51228

[B4] Bills K. L. Hayne C. Stein S. E. (2018). A field study on small accounting firm membership in associations and networks: implications for audit quality. *Account. Rev.* 93 73–96. 10.2308/accr-52003

[B5] Chang H. H. Wong K. H. (2018). Consumer psychological reactance to coalition loyalty program: price-consciousness as a moderator. *Serv. Bus.* 12 379–402. 10.1007/s11628-017-0353-6

[B6] Chen M. (2019). The impact of Expatriates’ cross-cultural adjustment on work stress and job involvement in the high-tech industry. *Front. Psychol.* 10:2228 10.3389/fpsyg.2019.02228 PMC6794360 31649581

[B7] Chhaochharia V. Grullon G. Grinstein Y. Michaely R. (2009). *Product Market Competition and Agency Conflicts: Evidence from the Sarbanes Oxley Law. Johnson School Research Paper Series. 2012.* Available online at: https://papers.ssrn.com/sol3/papers.cfm?abstract_id=1109225 (accessed November 23, 2015).

[B8] Choi C. Choi S. Mattila A. S. (2019). The role of reference prices in the lodging industry: the moderating effect of an individual’s psychological state. *J. Trav. Tourism Market.* 36 511–520. 10.1080/10548408.2019.1583626

[B9] Defond M. L. Zhang J. (2014). A review of archival auditing research. *J. Account. Econ.* 58 275–326. 10.1016/j.jacceco.2014.09.002

[B10] Finley A. Kim M. H. J. Lamoreaux P. T. Lennox C. S. (2018). Employee movements from audit firms to audit clients. *SSRN* 36 1999–2034. 10.1111/1911-3846.12494

[B11] Huang T. C. Lin Y. H. Hairston S. (2019). Is there an association between accounting firm ranks and audit quality? An examination of the top 100 accounting firms in China. *Int. J. Audit.* 23 204–230. 10.1111/ijau.12156

[B12] Krishnan G. V. Patatoukas P. N. Wang A. Y. (2019). Customer-base concentration: implications for audit pricing and quality. *J. Manag. Account. Res.* 31 129–152. 10.2308/jmar-52040

[B13] Kudryavtsev A. (2019). Psychological aspects of stock returns accompanied by high trading volumes. *J. Risk Control* 6 1–17.

[B14] Kuntari Y. Chariri A. Nurdhiana N. (2017). The effect of auditor ethics, auditor experience, audit fees and auditor motivation on audit quality. *SIJDEB* 1 203–218. 10.29259/sijdeb.v1i2.203-218

[B15] Kushlev K. Dwyer R. Dunn E. W. (2019). The social price of constant connectivity: smartphones impose subtle costs on well-being. *Curr. Dir. Psychol. Sci.* 28 347–352. 10.1177/0963721419847200

[B16] Lemmerer A. Menrad K. (2019). Acceptance of new food products: reference prices and psychological moderators of heterogeneous price effects. *J. Food Prod. Mark.* 25 713–733. 10.1080/10454446.2019.1647905

[B17] Li Y. Huang Z. Wu Y. J. Wang Z. (2019). Exploring how personality affects privacy control behavior on social networking sites. *Front. Psychol.* 10:1771 10.3389/fpsyg.2019.01771 PMC6685389 31417477

[B18] Mou S. H. Li Q. Yu L. P. (2014). Internal control, executive power and audit fees: an empirical study based on the data of non-financial listed companies in 2009-2012. *Audit Econ. Res.* 2014 42–51.

[B19] Obschonka M. Moeller J. Goethner M. (2019). Entrepreneurial passion and personality: the case of academic entrepreneurship. *Front. Psychol.* 9:2697 10.3389/fpsyg.2018.02697 PMC6335975 30687165

[B20] Pleman B. Park M. Han X. Price L. L. Bannuru R. R. Harvey W. F. (2019). Mindfulness is associated with psychological health and moderates the impact of fibromyalgia. *Clin. Rheumatol.* 38 1737–1745. 10.1007/s10067-019-04436-1 30644003 PMC6545163

[B21] Price M. Arditi R. Olezeski C. Mcmahon T. J. (2019). Psychological assessment and treatment of emerging adults exposed to complex trauma. *Evid Based Pract. Child Adolesc. Ment. Health* 4 273–295. 10.1080/23794925.2019.1618225

[B22] Rand K. Vallis M. Aston M. Price S. Piccinini-Vallis H. Rehman L. (2017). It is not the diet; it is the mental part we need help with.” A multilevel analysis of psychological, emotional, and social well-being in obesity. *Int. J. Qual. Stud. Health Wellbeing* 12:1306421 10.1080/17482631.2017.1306421 PMC5421368 28418818

[B23] Sari S. P. Diyanti A. A. Wijayanti R. (2019). The effect of audit tenure, audit rotation, audit fee, accounting firm size, and auditor specialization to audit quality. *REAKSI* 4 186–196. 10.23917/reaksi.v4i3.9492

[B24] Shen C. Chen M. Wang C. (2019). Analyzing the trend of O2O commerce by bilingual text mining on social media. *Comput. Hum. Behav.* 101 474–483. 10.1016/j.chb.2018.09.031

[B25] Shi X. W. Li Z. G. Liu Z. (2015). Research on analysts’ prediction and audit fees of listed companies from the perspective of information asymmetry theory. *Audit Econ. Res.* 030 39–48.

[B26] Sofia P. Avianti I. (2019). Influence of internal control activities and characteristics of audit committee on the quality of audit implementation by a public accounting firm. *J. Akuntansi* 23 97–112. 10.24912/ja.v23i1.465

[B27] Su Y. Han L. Wang J. Wang H. (2019). Quantum-behaved RS-PSO-LSSVM method for quality prediction in parts production processes. *Concurr. Comp. Pract. Exp.* 9:e5522 10.1002/cpe.5522

[B28] Sulistiyo H. Ghozali I. (2017). The role of religious control in dysfunctional audit behavior: an empirical study of auditors of public accounting firm in Indonesia. *JABR* 33 1047–1058. 10.19030/jabr.v33i5.10026

[B29] Tsao C. Y. Lee C. I. Shyu Y. W. (2017). Crossing of psychological price levels: the price dynamics and interaction between S&P500 index and index futures. *J. Behav. Financ.* 18 427–447. 10.1080/15427560.2017.1344675

[B30] Walsh J. Gibson A. Brown L. M. (2016). Peace of mind’s price tag: the psychological costs of financial stressors on older adults postdisaster. *Transl. Issues Psychol. Sci.* 2:408 10.1037/tps0000089

[B31] Wu Y. C. J. Wu T. (2017). A decade of entrepreneurship education in the Asia Pacific for future directions in theory and practice. *Manag. Decis.* 55 1333–1350. 10.1108/MD-05-2017-0518

[B32] Wu Y. J. Liu W.-J. Yuan C.-H. (2020). A mobile-based barrier-free service transportation platform for people with disabilities. *Comput. Hum. Behav.* 107:105776 10.1016/j.chb.2018.11.005

[B33] Wu Y. J. Song D. (2019). Gratifications for social media use in entrepreneurship courses: learners’ perspective. *Front. Psychol* 10:1270 10.3389/fpsyg.2019.01270 PMC6555126 31214081

[B34] Yu J. (2020). Exploring the role of healthy green spaces, psychological resilience, attitude, brand attachment, and price reasonableness in increasing hotel guest retention. *Int. J. Environ. Res. Public Health* 17:133 10.3390/ijerph17010133 PMC6981477 31878113

